# Supplementary figures and images for: Medication use is associated with distinct microbial features in anxiety and depression
Source: Mol Psychiatry. 2025 Jan 10;30(6):2545–57. doi: 10.1038/s41380-024-02857-2 (PMC12092254; doi:10.1038/s41380-024-02857-2)

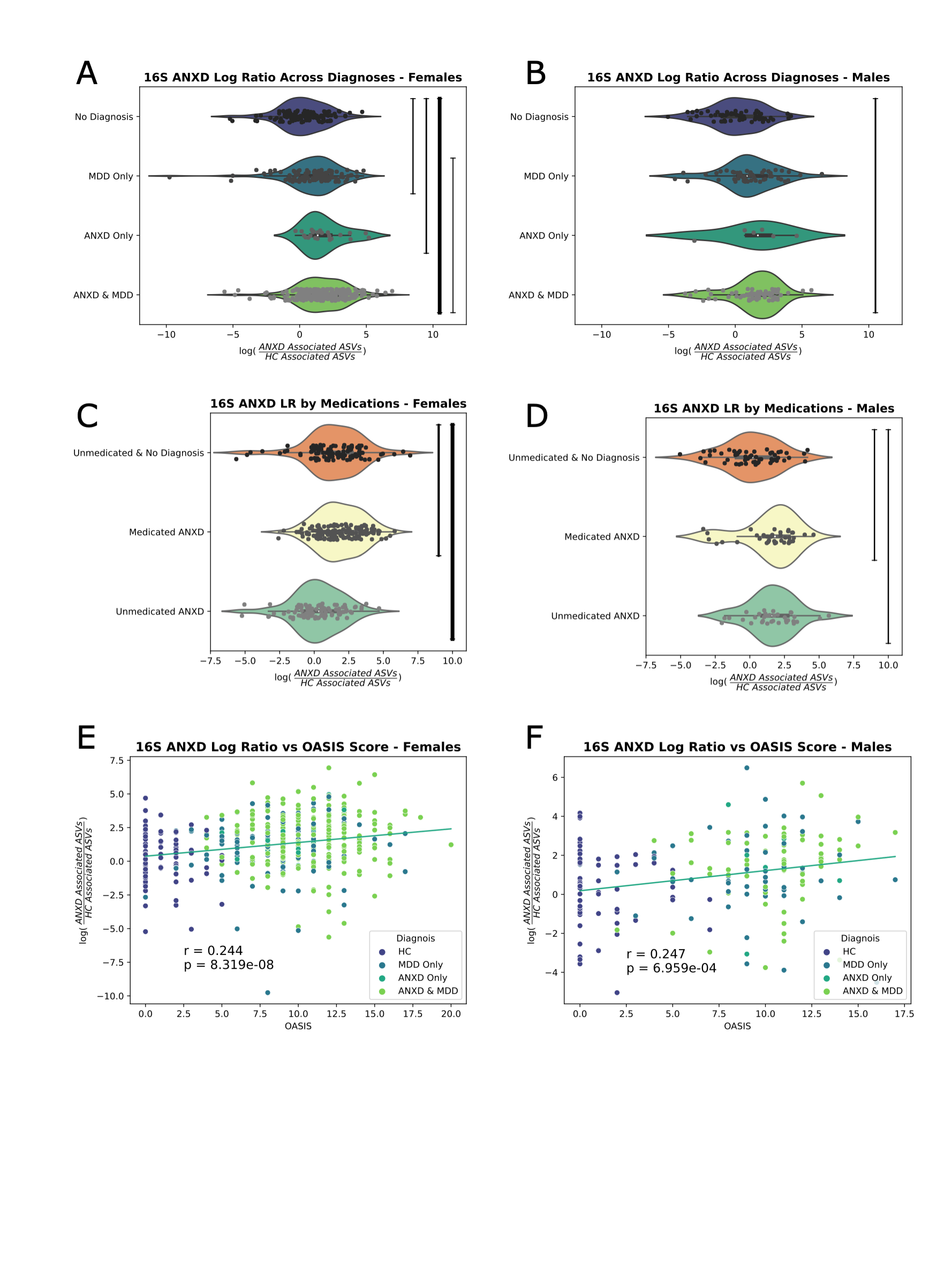

Supplement: Supplementary file 1 — Figure S1 [file 41380_2024_2857_MOESM1_ESM.png]

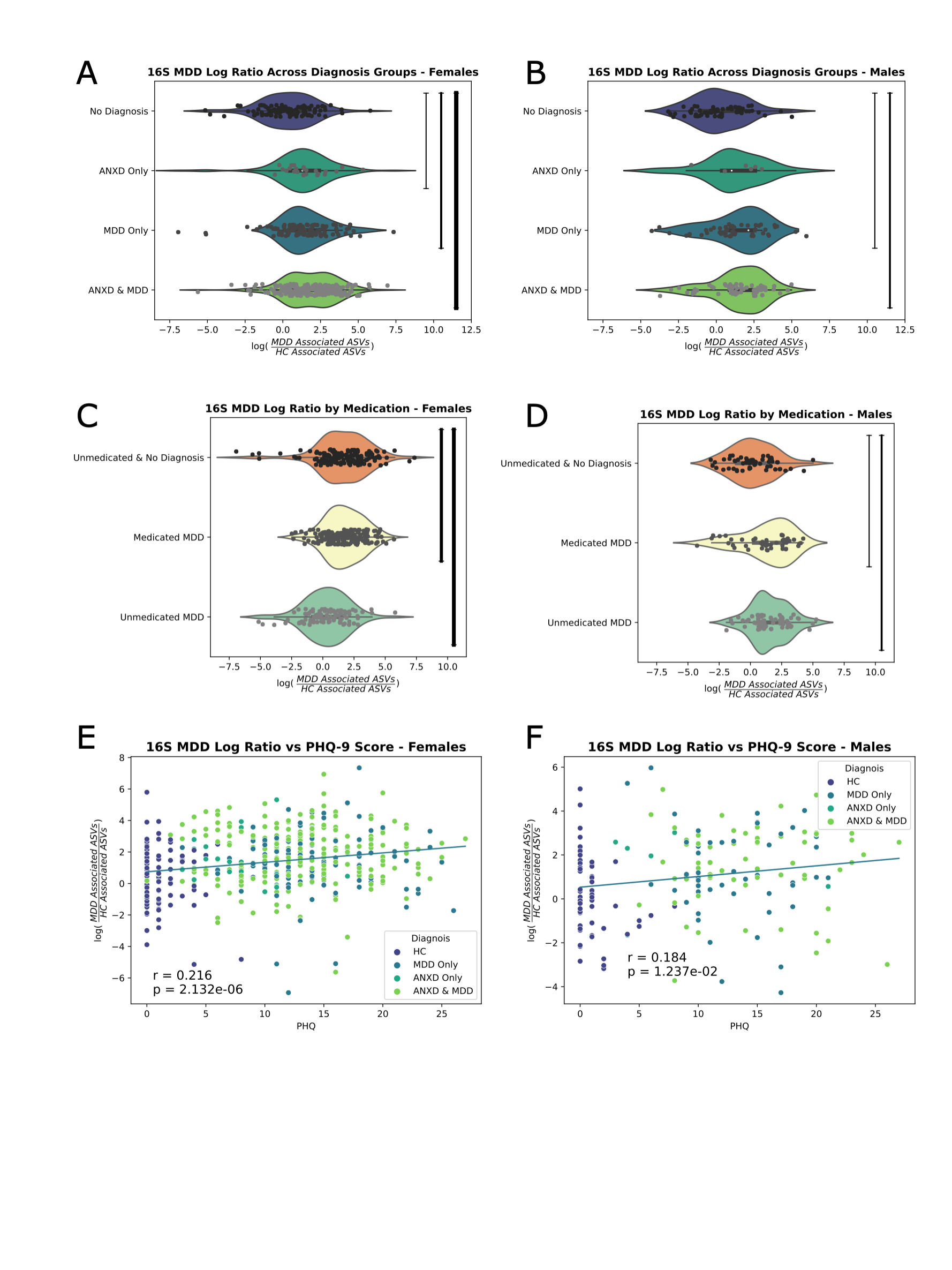

Supplement: Supplementary file 2 — Figure S2 [file 41380_2024_2857_MOESM2_ESM.png]

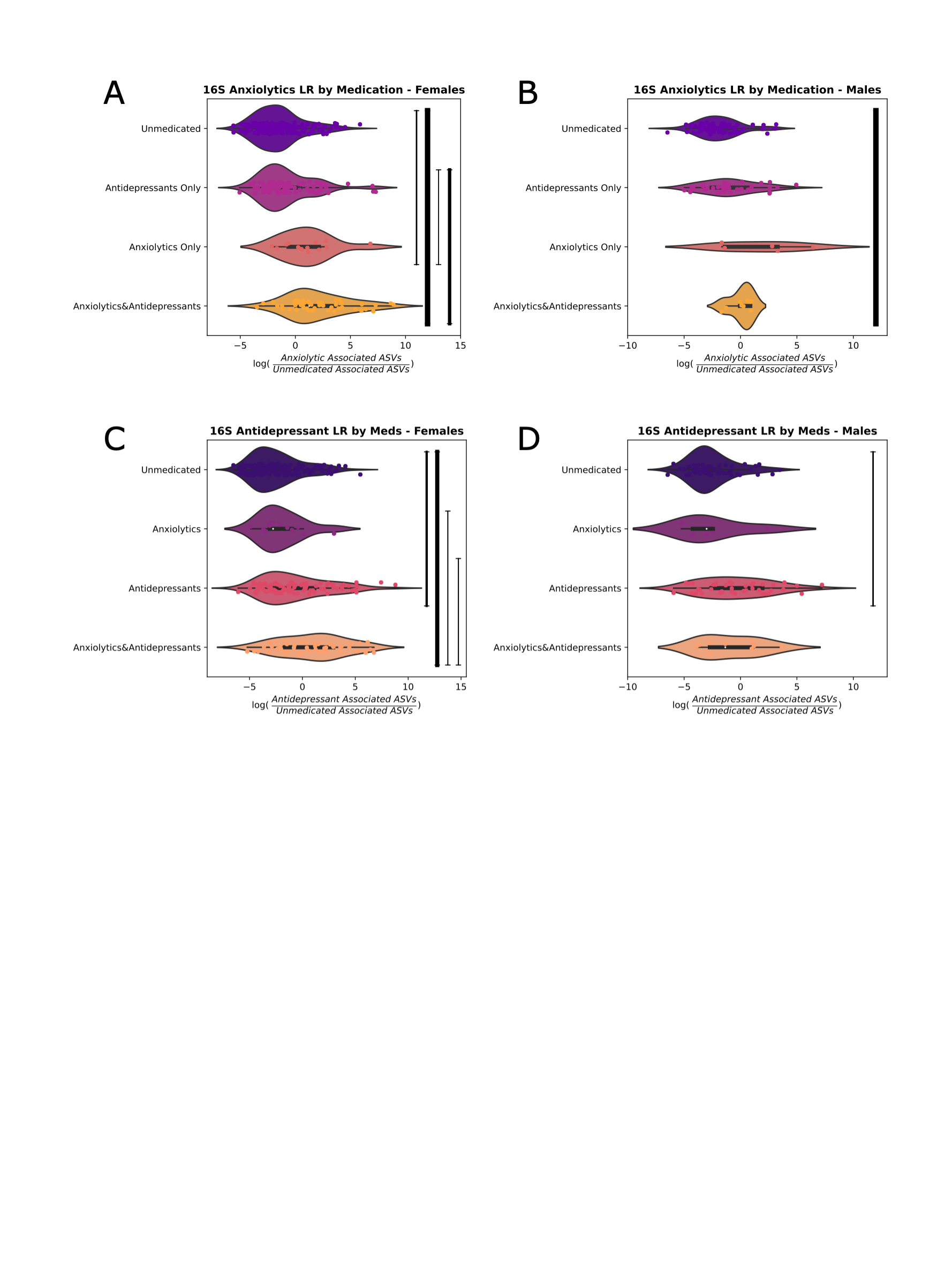

Supplement: Supplementary file 3 — Figure S3 [file 41380_2024_2857_MOESM3_ESM.png]

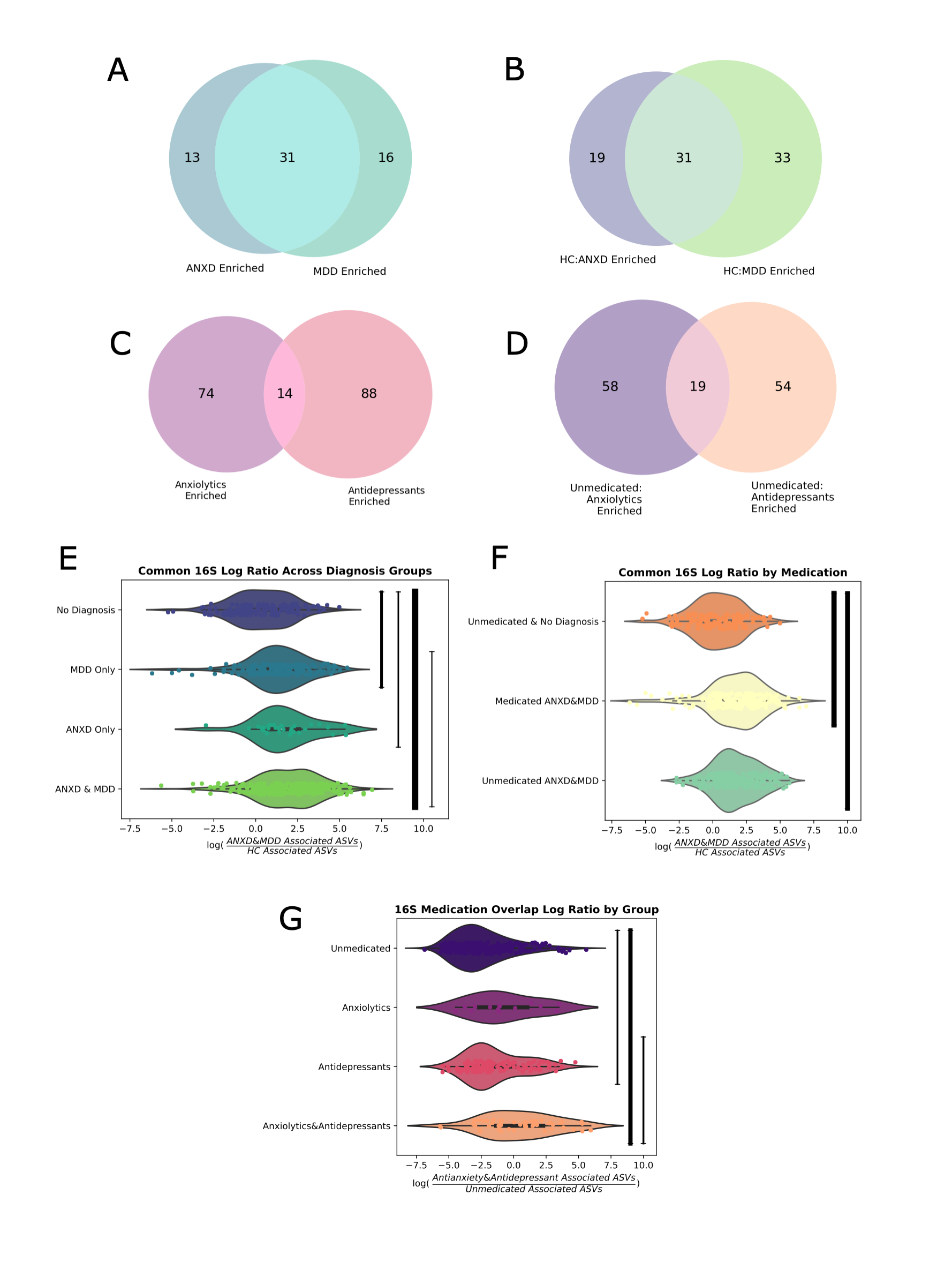

Supplement: Supplementary file 4 — Figure S4 [file 41380_2024_2857_MOESM4_ESM.png]

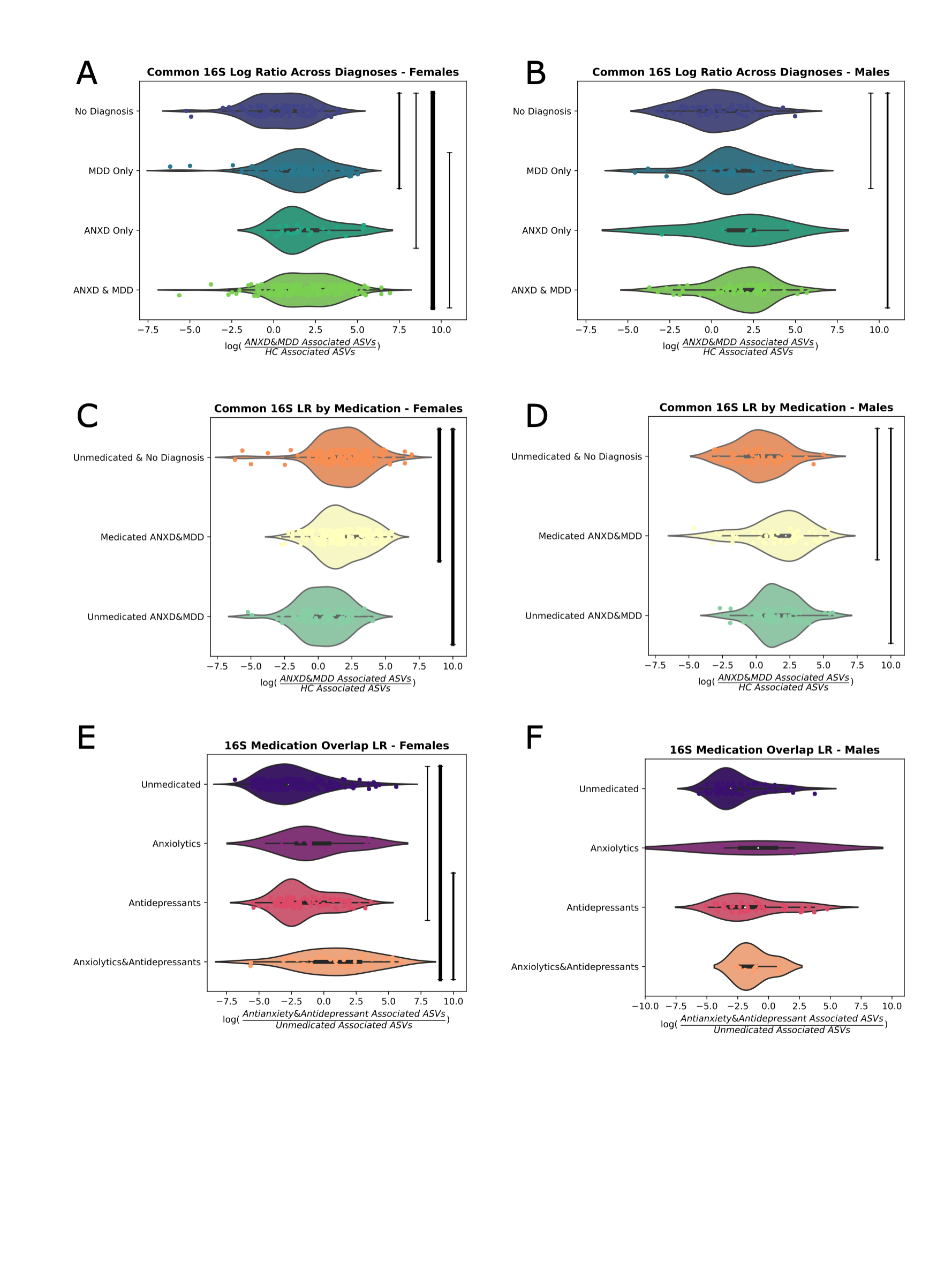

Supplement: Supplementary file 5 — Figure S5 [file 41380_2024_2857_MOESM5_ESM.png]

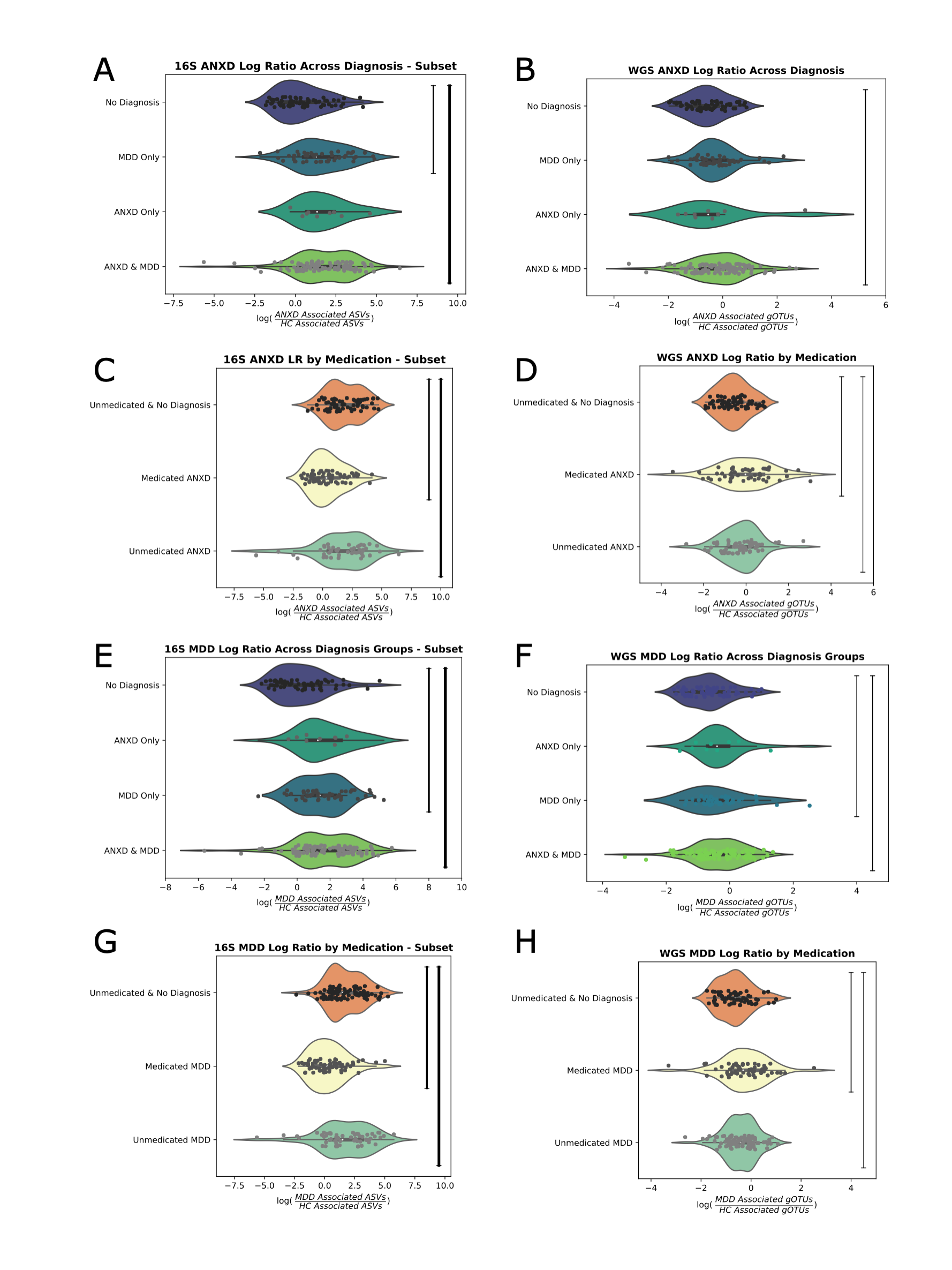

Supplement: Supplementary file 6 — Figure S6 [file 41380_2024_2857_MOESM6_ESM.png]

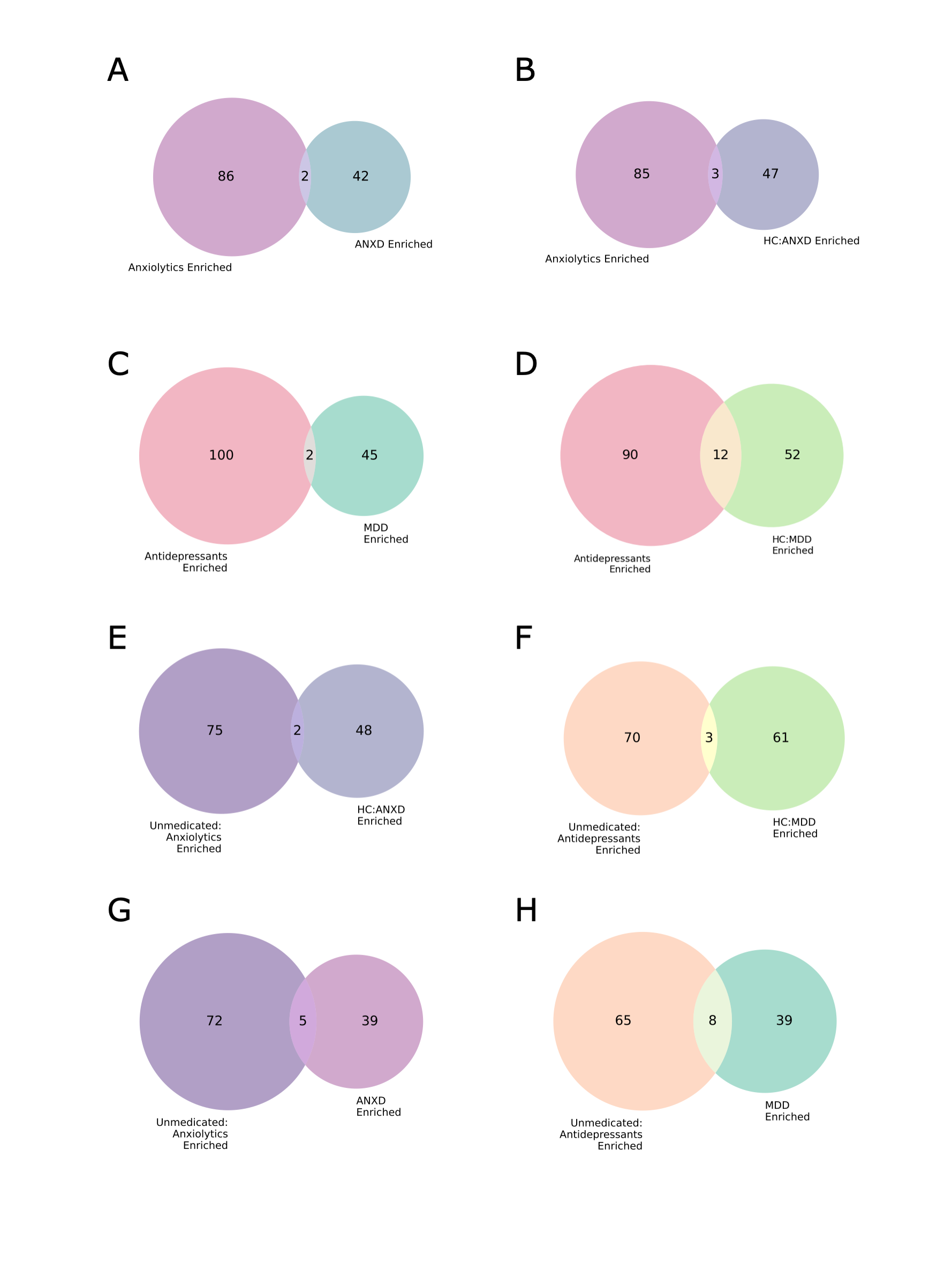

Supplement: Supplementary file 7 — Figure S7 [file 41380_2024_2857_MOESM7_ESM.png]

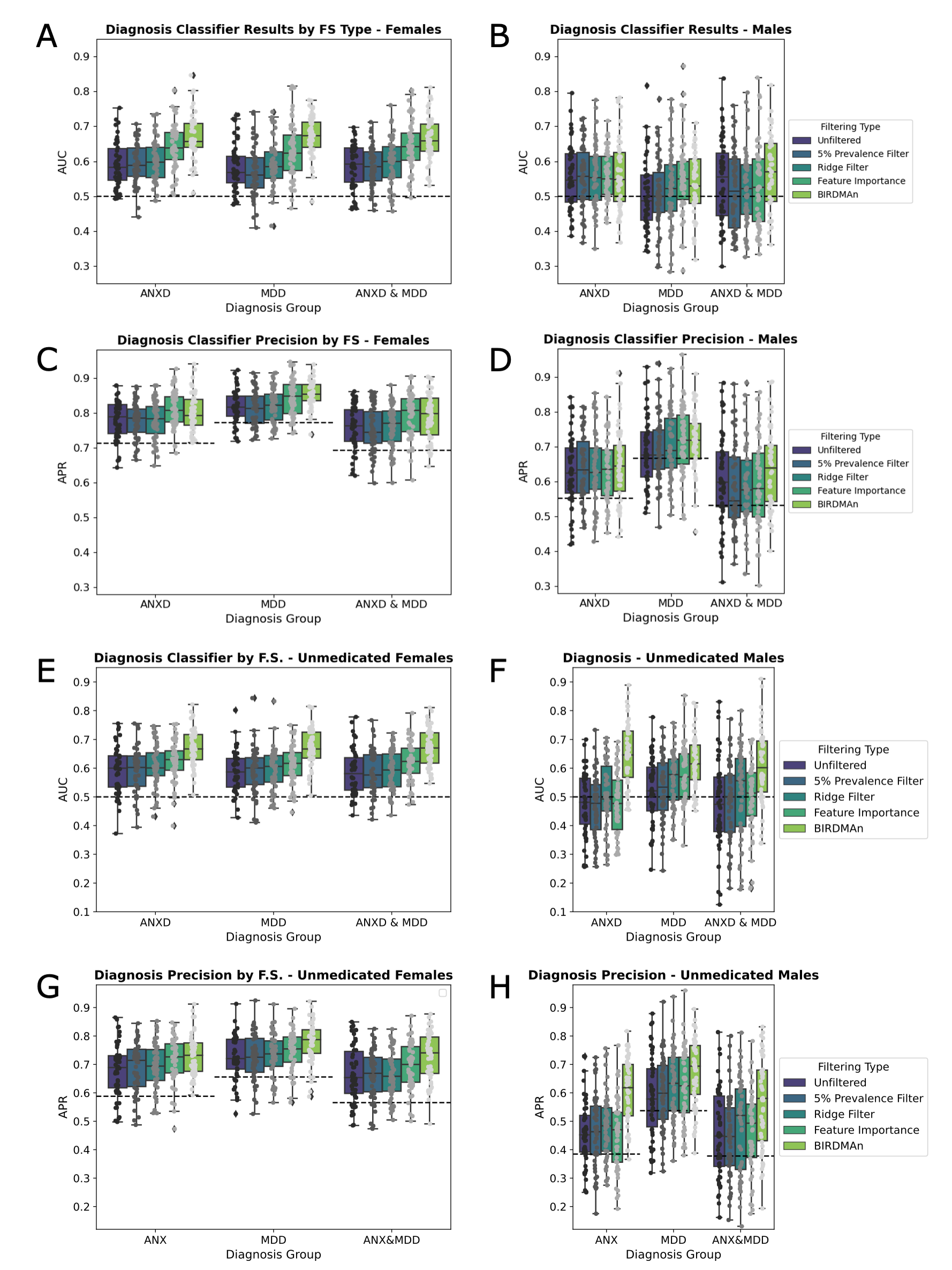

Supplement: Supplementary file 8 — Figure S8 [file 41380_2024_2857_MOESM8_ESM.png]

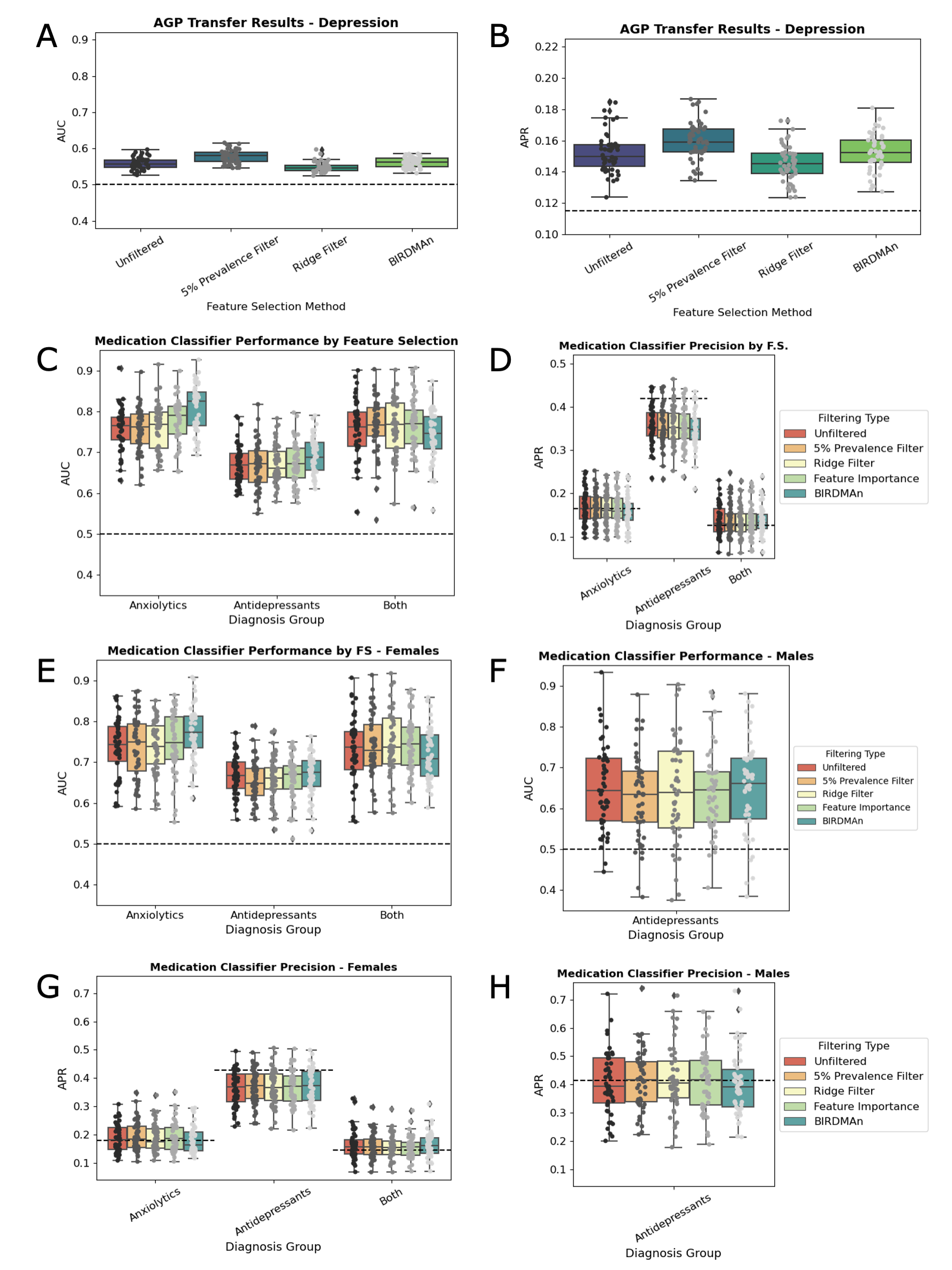

Supplement: Supplementary file 9 — Figure S9 [file 41380_2024_2857_MOESM9_ESM.png]
