# Supplementary material for: Medication use is associated with distinct microbial features in anxiety and depression
Source: Mol Psychiatry. 2025 Jan 10;30(6):2545–57. doi: 10.1038/s41380-024-02857-2 (PMC12092254; doi:10.1038/s41380-024-02857-2)
Supplement: Supplementary file 10 — Supplementary Info [file 41380_2024_2857_MOESM10_ESM.docx]

**Figure S1 - Sex stratified validation of microbes enriched in ANXD.** Log ratios were created comparing the relative abundance of all credible microbes associated with ANXD to all credible microbes associated with HC. Thickness of the line is correlated with the negative log of the p-value between the two groups. **(A)** Log ratios displayed by diagnosis - females. **(B)** Log ratios displayed by diagnosis - males. **(C)** Log ratios displayed by medication status - females. **(D)** Log ratios displayed by medication status - males. **(E)** Scatterplot between ANXD log ratio and OASIS score - females. **(F)** Scatterplot between ANXD log ratio and OASIS score - males.

**Figure S2 - Sex stratified validation of microbes enriched in MDD.** Log ratios were created comparing the relative abundance of all credible microbes associated with MDD to all credible microbes associated with HC. Thickness of the line is correlated with the negative log of the p-value between the two groups. **(A)** Log ratios displayed by diagnosis - females. **(B)** Log ratios displayed by diagnosis - males. **(C)** Log ratios displayed by medication status - females. **(D)** Log ratios displayed by medication status - males. **(E)** Scatterplot between MDD log ratio and PHQ-9 score - females. **(F)** Scatterplot between MDD log ratio and PHQ-9 score - males.

**Figure S3 - Sex stratified validation of microbes enriched in anxiolytic and/or antidepressant use.** Anxiolytic log ratios were created comparing the relative abundance of all credible microbes associated with anxiolytics to all credible microbes associated with no medication usage. **(A)** Anxiolytic log ratios displayed by medication group - females. **(B)** Anxiolytic log ratios displayed by medication group - males. Antidepressant log ratios were created comparing the relative abundance of all credible microbes associated with antidepressants to all credible microbes associated with no medication usage. **(C)** Antidepressant log ratio displayed by medication status - females. **(D)** Antidepressant log ratios displayed by medication status - males.

**Figure S4 - ANXD and MDD have similar effects on gut microbiota; anxiolytics and antidepressants have more distinct effects. (A)** Venn diagram showing overlap in the credible microbes associated with ANXD and MDD. **(B)** Venn diagram showing overlap in the credible microbes associated with HC relative to ANXD and HC relative to MDD. **(C)** Venn diagram showing overlap in the credible microbes associated with anxiolytics and antidepressants. **(D)** Venn diagram showing overlap in the credible microbes associated with unmedicated participants relative to those on anxiolytics and unmedicated participants relative to those on antidepressants. **(E)** A log ratio was created using the microbes that were credibly associated with both ANXD and MDD and the microbes associated with HC relative to both ANXD and MDD. This log ratio was elevated in participants with MDD only, ANXD only, and ANXD and MDD relative to HC. It was also enriched in participants with ANXD and MDD relative to MDD alone. **(F)** This “common” credible log ratio was also enriched in both unmedicated and medicated participants with ANXD and MDD relative to unmedicated participants. **(G)** Another log ratio was created using the microbes that were credibly associated with both anxiolytics and antidepressants and the microbes associated with unmedicated participants relative to both anxiolytics and antidepressants.

**Figure S5 - Sex stratified validation of microbes enriched in both ANXD and MDD.** A log ratio was created using the microbes that were credibly associated with both ANXD and MDD and the microbes associated with HC relative to both ANXD and MDD. **(A)** Common log ratio displayed by diagnosis - females. **(B)** Common log ratio displayed by diagnosis - males. **(C)** Common log ratio displayed by medication status - females. **(D)** Common log ratio displayed by medication status - males. Another log ratio was created using the microbes that were credibly associated with both Anxiolytics and Antidepressants and the microbes associated with unmedicated participants relative to both medication types. **(E)** Common log ratio displayed by medication group - females. **(F)** Common log ratio displayed by medication group - males.

**Figure S6 - Smaller differences in enriched microbes in metagenomic data may be explained by the differences in the subset themselves. (A)** The log ratio of microbes credibly associated with ANXD relative to microbes credibly associated with HC displayed only in participants who have a matching sample sequenced by WGS as well. In this subset, there is a significant difference in participants with MDD only and participants with ANXD and MDD relative to HC. **(B)** We used nearest neighbors on the Greengenes2 (GG2) phylogenetic tree to transfer our credibly associated microbes with 16S data to WGS data. This WGS log ratio for ANXD was enriched only in participants with both ANXD and MDD. **(C)** The ANXD-specific log ratio was significantly lower in medicated participants with ANXD and significantly higher in unmedicated participants with ANXD relative to unmedicated HC. **(D)** The WGS ANXD-specific log ratio was significantly higher in both medicated and unmedicated participants with ANXD relative to unmedicated HC. **(E)** The log ratio of microbes credibly associated with MDD relative to microbes credibly associated with HC displayed only in participants who have a matching sample sequenced by WGS as well. In this subset, there is a significant difference in participants with MDD only and participants with ANXD and MDD relative to HC. **(F)** We used nearest neighbors on the GG2 phylogenetic tree to transfer our credibly associated microbes with 16S data to WGS data. This WGS log ratio for MDD was enriched in participants with MDD alone and those with ANXD and MDD relative to HC. **(G)** The MDD-specific log ratio was significantly lower in medicated participants with MDD and significantly higher in unmedicated participants with MDD relative to unmedicated HC. **(H)** The WGS MDD-specific log ratio was significantly higher in both medicated and unmedicated participants with MDD relative to unmedicated HC.

**Figure S7 - Little overlap in microbes enriched in medications and those enriched in psychiatric disease. (A)** Venn diagram showing minimal overlap in the credible microbes associated with ANXD and anxiolytics. **(B)** Venn diagram showing minimal overlap in the credible microbes associated with HC relative to ANXD and anxiolytics. **(C)** Venn diagram showing minimal overlap in the credible microbes associated with MDD and antidepressants. **(D)** Venn diagram showing some overlap in the credible microbes associated with HC relative to MDD and antidepressants. **(E)** Venn diagram showing minimal overlap in the credible microbes associated with HC relative to ANXD and unmedicated participants relative to those on anxiolytics. **(F)** Venn diagram showing minimal overlap in the credible microbes associated with HC relative to MDD and unmedicated participants relative to those on antidepressants. **(G)** Venn diagram showing minimal overlap in the credible microbes associated with ANXD and unmedicated participants relative to those on anxiolytics. **(H)** Venn diagram showing some overlap in the credible microbes associated with MDD and unmedicated participants relative to those on antidepressants.

**Figure S8 - Sex stratified performance of Random Forest Classifiers for ANXD/MDD Diagnosis.** **(A)** Area under the receiver operating characteristic curve (AUC) is displayed for a diagnosis classifier distinguishing ANXD diagnosis, MDD diagnosis, or ANXD and MDD diagnosis from HC for females in the study. Four types of feature selection were applied in addition to the raw data. **(B)** Random Forest Classifier was applied to only male participants. **(C)** Average precision (APR) scores were also calculated across the different classifications and feature selection methods; only females are shown in this plot. **(D)** APR scores in the male subset. (**E)** AUC displayed for only unmedicated females. **(F)** AUC displayed for only unmedicated males. (**G)** APR scores for only unmedicated females. **(H)** APR scores for only unmedicated males.

**Figure S9 - Random Forest Classifiers demonstrate high accuracy but low precision when applied to American Gut cohort and medication use, even when sex-stratified.** We used a Random Forest Classifier to distinguish AG participants who self-reported a MDD diagnosis from those who self-reported no diagnosis. **(A)** Area under the receiver operating characteristic curve (AUC) for the AGP diagnosis classifier. **(B)** Average precision scores (APR) for the AGP diagnosis classifier. **(C)** AUCs for a medication classifier across the cohort. **(D)** APRs for the medication classifier. **(E)** AUCs for a medication classifier across females. **(F)** AUCs for an antidepressant classifier for males. **(G)** APR scores for a medication classifier across females. **(H)** APR scores for an antidepressant classifier for males.

**Table S1 - Alpha diversity statistics for the Tulsa-1000 cohort.**

**Table S2 - Beta diversity statistics for the Tulsa-1000 cohort without Adonis results.**

**Table S3 - Adonis beta diversity statistics for the Tulsa-1000 cohort.**

**Table S4 - Sex-stratified beta diversity significance results for the Tulsa-1000 cohort.**

**Table S5 - BIRDMAn differential abundance statistics for the Tulsa-1000 cohort.**

**Table S6 - Random Forest Classifier results.**

**Table S7 - Female specific Random Forest Classifier results.**

**Table S8 - Male specific Random Forest Classifier results.**
